# Supplementary material for: Wetting ridge assisted programmed magnetic actuation of droplets on ferrofluid-infused surface
Source: Nat Commun. 2021 Dec 8;12:7136. doi: 10.1038/s41467-021-27503-1 (PMC8654979; doi:10.1038/s41467-021-27503-1)
Supplement: Supplementary file 1 — Supplementary Information [file 41467_2021_27503_MOESM1_ESM.pdf]

Supplementary Information for

Wetting ridge assisted programmed magnetic actuation of droplets on ferrofluid-infused surface

Jianqiang Zhang[1], Xuejiao Wang[1], Zhaoyue Wang[1], Shangfa Pan[2], Bo Yi[1], Liqing Ai[1], Jun Gao[2] ✉, Frieder Mugele[3] ✉, Xi Yao[1,4] ✉

[1] Department of Biomedical Sciences, City University of Hong Kong, Hong Kong, P. R. China

[2] Qingdao Institute of Bioenergy and Bioprocess Technology, Chinese Academy of Sciences, Qingdao, 266101 P. R. China

[3] Physics of Complex Fluids, MESA+ Institute for Nanotechnology, University of Twente, P.O. Box 217, Enschede, 7500AE the Netherlands

[4] Shenzhen Research Institute, City University of Hong Kong, Shenzhen 518075 P. R. China

✉E-mail: jun.gao@qibebt.ac.cn; f.mugele@utwente.nl; xi.yao@cityu.edu.hk

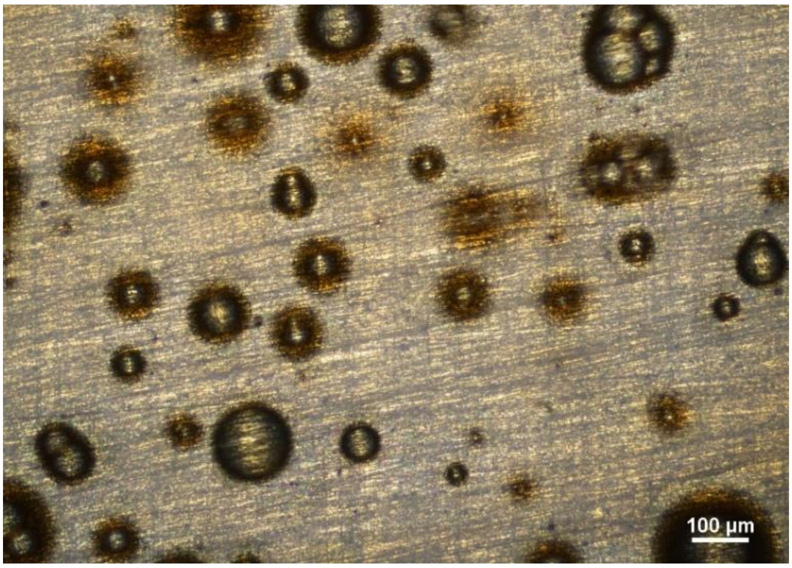

Supplementary Fig. 1 | Magnified view of some microdroplets sprayed on the slippery surface in Fig. 1 in the main manuscript. The minimum size of the droplets (bright spots) is ~10^1 μm. However, accurate estimation of the size is challenging due to the existence of the wetting ridge (black circles around the bright spots). Scale bar: 100 μm.

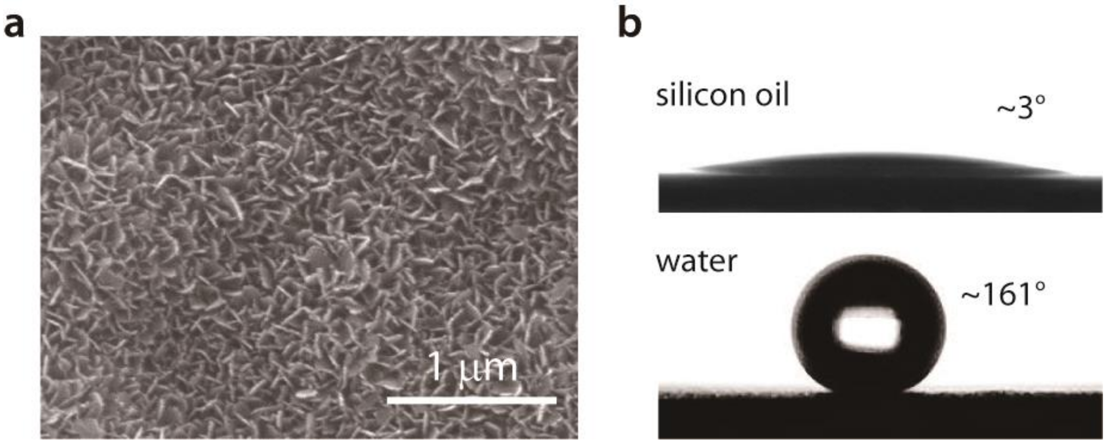

Supplementary Fig. 2 | Characterization of superhydrophobic aluminum substrate. a, SEM image of the nanostructured substrate. **b**, contact angles of the silicone oil and water on the substrate, suggesting the superoleophobic and superhydrophobic property, thus favoring the infusing of silicon oil.

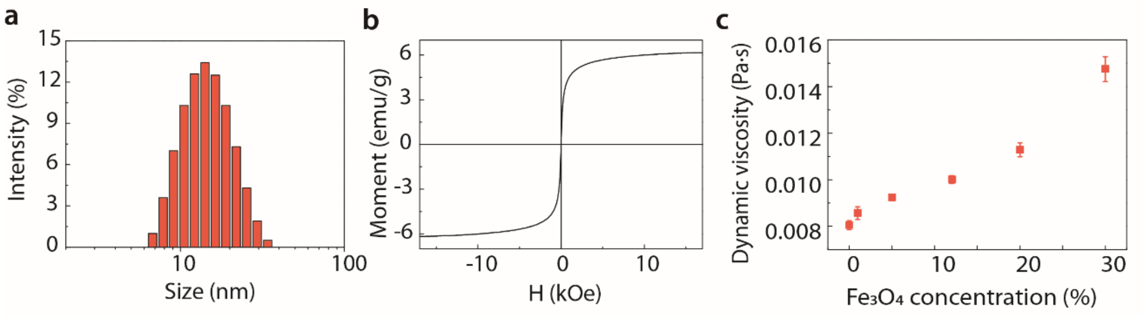

Supplementary Fig. 3 | Properties of the ferrofluid used in the experiment. (A) The particle size distribution of Fe_3O_4 nanoparticles. (B) Magnetization curve of the used ferrofluid (12 v/v% Fe_3O_4 nanoparticles). The saturation magnetic susceptibility is 6.2 emu/g. (c) Viscosity of the ferrofluid in response to the volumetric concentration of the Fe_3O_4 nanoparticle.

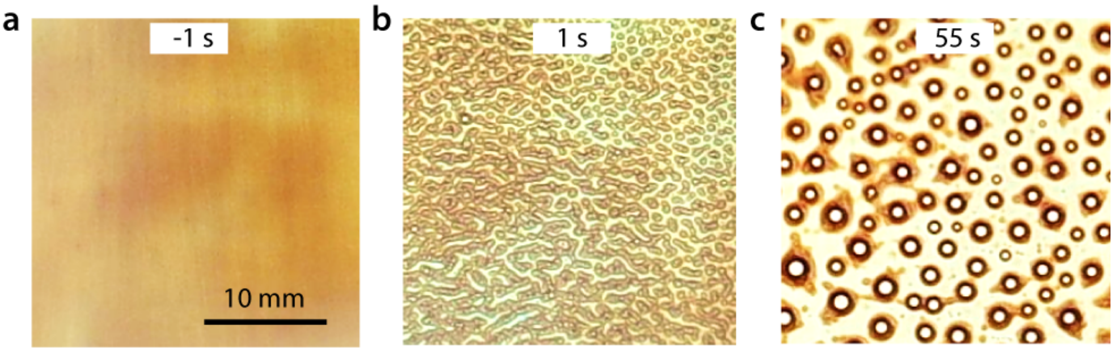

Supplementary Fig. 4 | Random coalescence of droplets without WRAP actuation. -1 s means 1 s before microdroplets were sprayed.

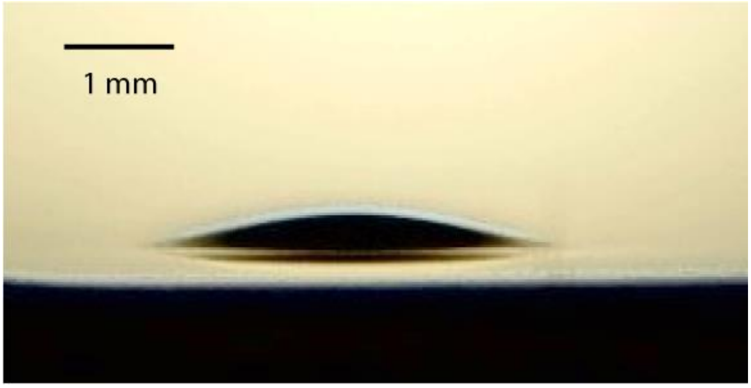

Supplementary Fig. 5 | Side-view optical image of a ferrofluid cusp above the activated electromagnet.

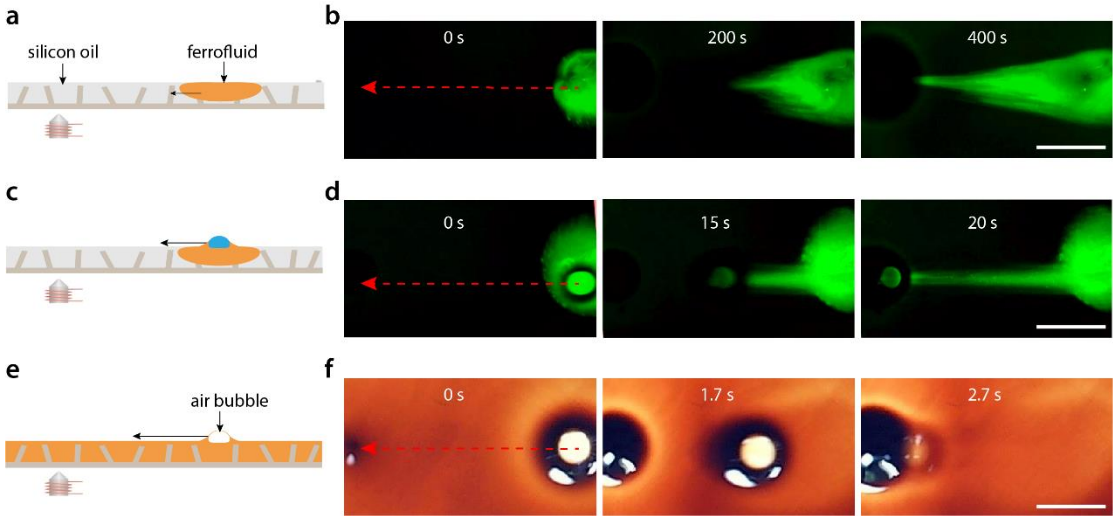

Supplementary Fig. 6 | Importance of the wetting ridge for the actuation. a, Confocal fluorescence snapshots showing the slow driving of a drop of infused ferrofluid, suggesting that the movement of the ferrofluid (dyed) infused in the slippery substrate (infused with silicon oil) contributes insignificantly to the droplet actuation. **b**, When a water droplet is placed on the infused ferrofluid, the droplet can be quickly driven to the magnet, suggesting the critical role of the wetting ridge. **c**, When the water droplet is replaced with an air bubble of similar size, it can also be quickly driven to the magnet, again validating the importance of the ridge. The air bubble is driven faster than the water droplet, because air is much more light-weight. All scale bars are 5 mm.

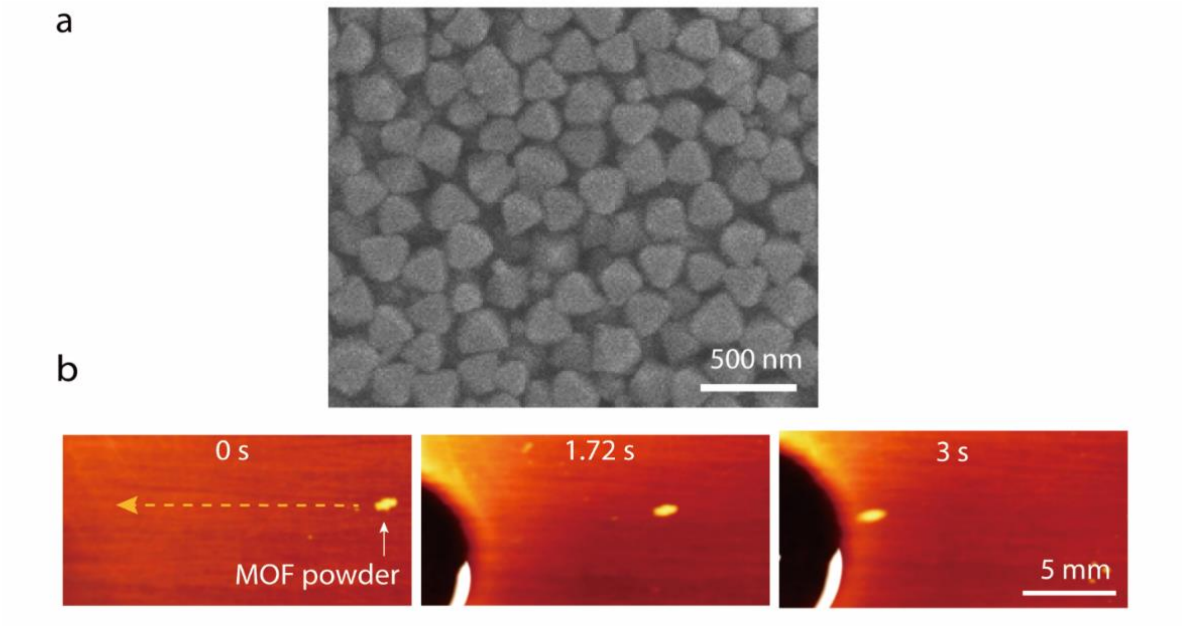

Supplementary Fig. 7 | Actuation of metal-organic-framework (MOF) powder, showing that WRAP can actuate solid samples. (a) SEM image of the synthesized MOF. (b) Optical images of the actuation process.

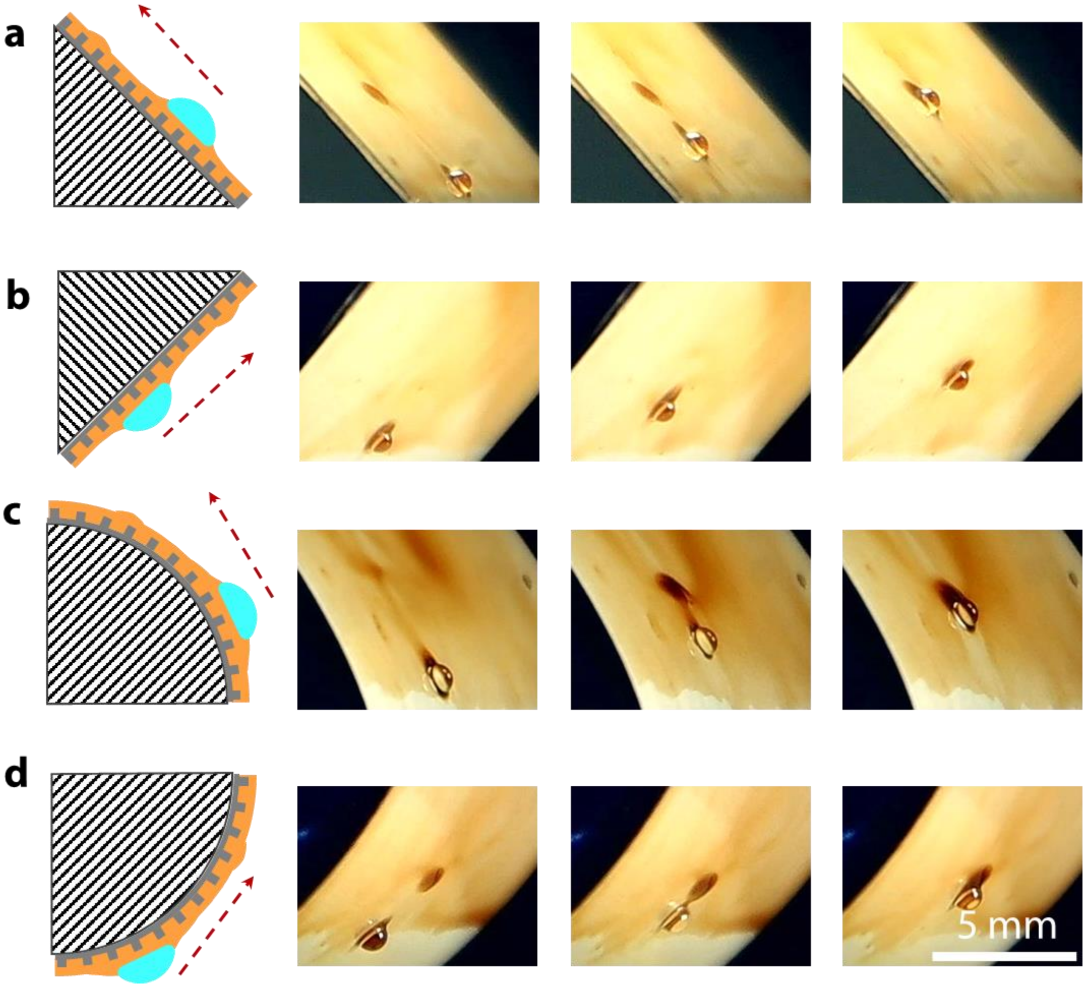

Supplementary Fig. 8 | Various actuation against gravity. The droplet can be transported against gravity, such as on 45° tilted planar surface (**a, b**) or curvy surface (**c, b**).

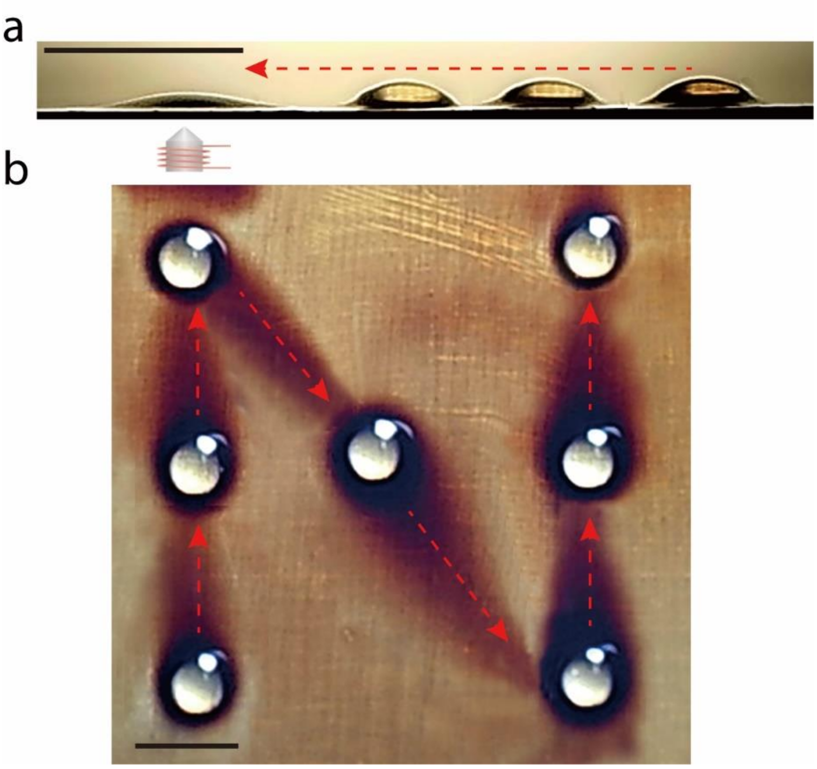

Supplementary Fig. 9 | Actuation of silicone oil droplet on fluorocarbon-based ferrofluid. (a) Side view of droplet movement along a straight line. (b) Programmed actuation of droplet along a N-shape path. Scale bars in both images are 2 mm.

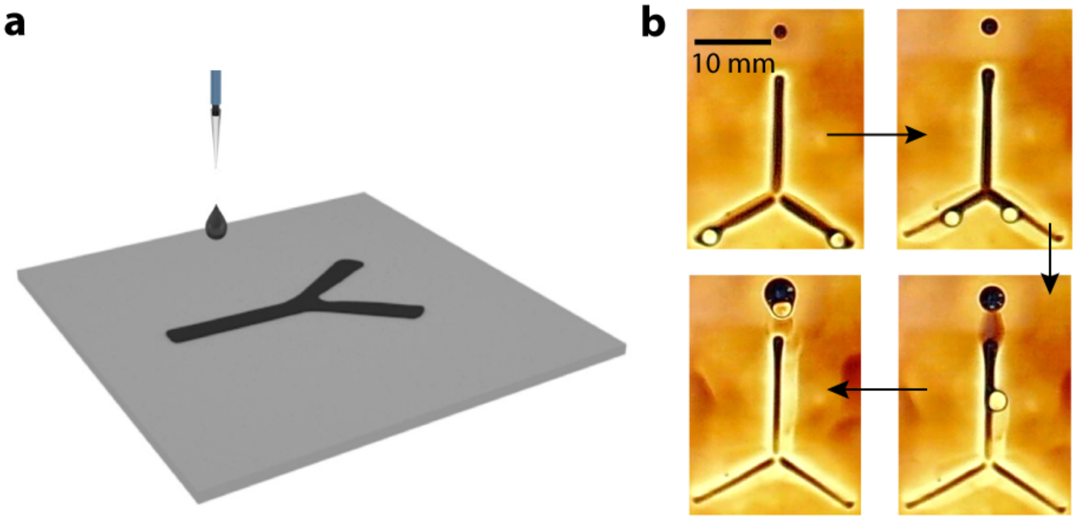

Supplementary Fig. 10 | Printing field modulation pattern. Using a home-built device, we can print ferromagnetic pattern (a), made of Fe_3O_4 nanoparticles in PDMS, on the substrate, to guide droplet transport (b).

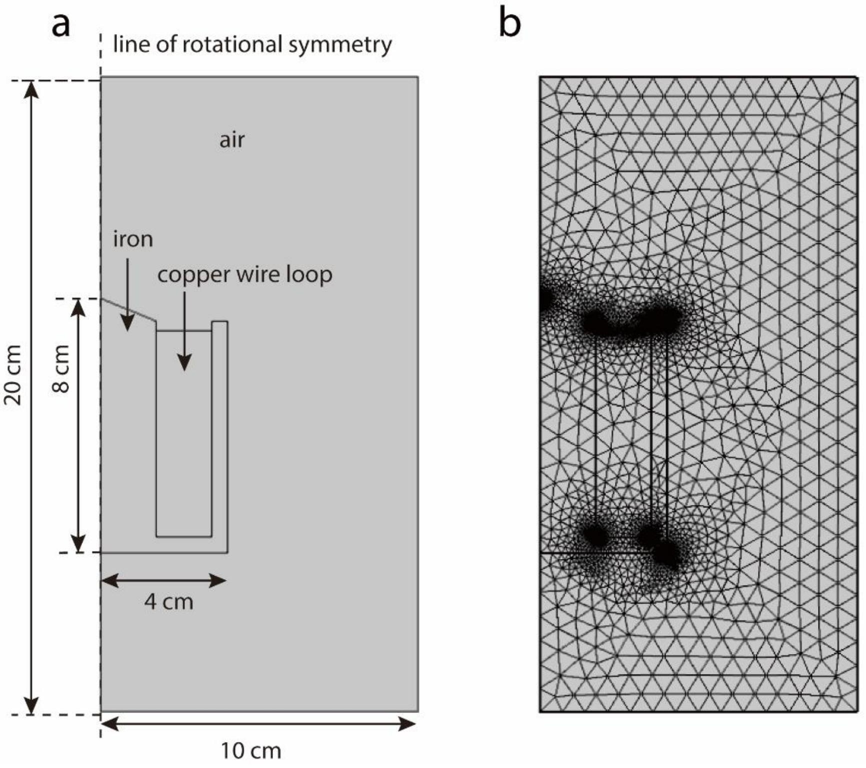

Supplementary Fig. 11 | Simulation of the magnetic field. a, the simulation model of the electromagnet. The dimensions of the copper wire loop and the iron are consistent with the real magnet used in our study. **b**, the mesh for the simulation. Note that the ferromagnetic pattern layer, which is on top of the electromagnet, is not shown here for clarity. This layer is simulated using the same dimensions and materials as used in experiments.

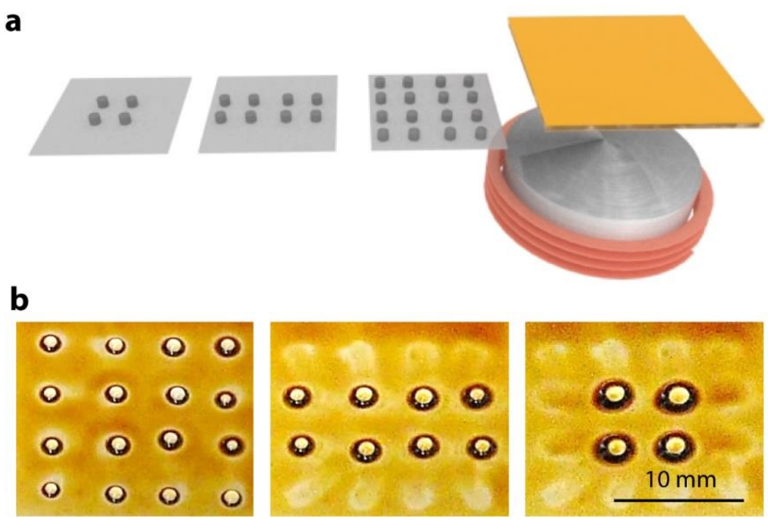

Supplementary Fig. 12 | Switching field modulation pattern to guide sequential droplet actuation. a, Schematic illustration of the process. Three patterns were sequentially inserted between the electromagnet and the slippery substrate. **b**, as a result, sprayed microdroplets sequentially coalesce to form different patterns.

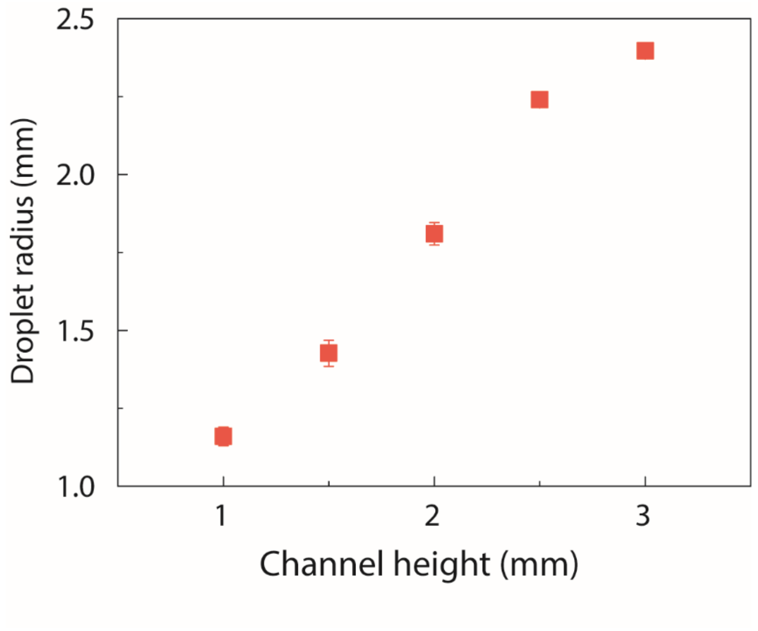

Supplementary Fig. 13 | Control of the droplet radius by varying the channel height. Fixed channel length: 3 mm. Fixed width:1 mm. Error bars represent the standard deviation (n= 3).

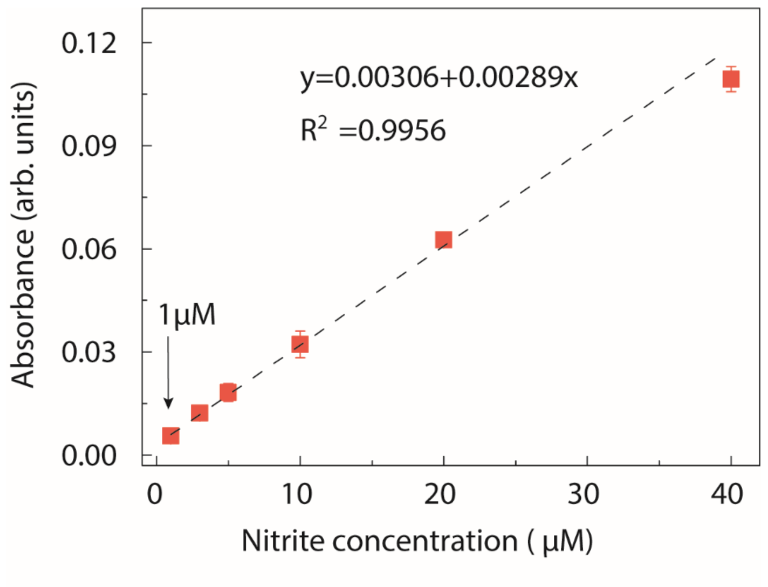

Supplementary Fig. 14 | Detection of low concentration nitrite. Nitrite with initial concentration ranging from 1 μM to 40 μM was also analyzed. The results show that 1 μM can quantitatively detected. Error bars represent the standard deviation (n= 3).

Supplementary Note 1

Surface instability analysis of the ferrofluid cusp

Ferrofluid typically experiences surface instability in strong magnetic field. In our experiment, such behavior was not observed, because of the low magnetic susceptibility and the low magnetic field strength. The surface instability is determined by the competition between the Laplace pressure of the droplet which tends to keep the droplet shape, and the magnetic pressure which tends to deform the droplet. Thus, the "magnetocapillary" number (*Mc*), *i.e.* the ratio of magnetic pressure to Laplace pressure, $Mc = \frac{\mathbf{B}^2}{\mu} R/2\gamma$ can be used to access this competition. Here **B** is the magnetic field, μ the magnetic permeability, *R* the ferrofluid droplet radius, and γ the ferrofluid/air interfacial tension. μ is related the magnetic susceptibility χ by $\mu=\mu_0(1+\chi)$. *Mc* is therefore calculated to be around 10^{-2}- 10^{-1}, meaning that the Laplace is dominating, explaining why surface instability was not observed.

Supplementary Table 1 Initial spreading coefficients

Interfaces Tension of water-air ($\gamma_{12,}$ mN/m)	Interfaces Tension of ferrofluid-air ($\gamma_{31,}$ mN/m)	Interfaces Tension of ferrofluid-water ($\gamma_{31,}$ mN/m)	Initial spreading coefficients ($S_i=\gamma_{12}-(\gamma_{31}+\gamma_{32})$)
72.05±0.31	1% ferrofluids -Air (10.57±0.29)	1% ferrofluids – Water (23.28±0.41)	>0
72.05±0.31	5% ferrofluids -Air (10.36±0.23)	5% ferrofluids-Water (22.94±0.36)	>0
72.05±0.31	12% ferrofluids -Air (8.33±0.25)	12% ferrofluids-Water (19.29±0.59)	>0

Interfacial tension was calculated with pendant drop method. The concentration of ferrofluid refers to the volumetric concentrations of Fe_3O_4 nanoparticles. Note: in our experiments except Fig. 1e, 12 v/v% ferrofluid was used.
