# Supplementary material for: Wetting ridge assisted programmed magnetic actuation of droplets on ferrofluid-infused surface
Source: Nat Commun. 2021 Dec 8;12:7136. doi: 10.1038/s41467-021-27503-1 (PMC8654979; doi:10.1038/s41467-021-27503-1)
Supplement: Supplementary file 3 — Description of Additional Supplementary Files [file 41467_2021_27503_MOESM3_ESM.pdf]

Description of Additional Supplementary Files

File Name: Supplementary Movie 1
Description: **Actuation of micro-sized droplets.** To demonstrate the basic functionality of WRAP, we first sprayed micro-sized droplets on the ferrofluid-infused surface, and turned on the electromagnet beneath the center of the surface. All droplets were actuated, driven to the center, and coalesced to form a single droplet.

File Name: Supplementary Movie 2
Description: **A typical actuation process of a single droplet.** A single droplet was deposited on the surface and driven to the magnet with WRAP. The position of the magnet was the final position of the droplet.

File Name: Supplementary Movie 3
Description: **Actuation of a droplet inside a straight channel.** A droplet was injected into a straight channel with ferrofluid-infused surface, and actuated with WRAP, showing the potential application of WRAP in closed microfluidics.

File Name: Supplementary Movie 4
Description: **Actuation of a droplets inside a curved channel.** A droplet was injected into a curved channel with ferrofluid-infused surface, and actuated with WRAP, further confirming the potential application of WRAP in closed microfluidics.

File Name: Supplementary Movie 5
Description: **Actuation of a droplet outside of a rod.** A droplet was deposited on a rod with ferrofluid-infused surface and placed upside down. The droplet adhered to the rod, but can still be actuated in the horizontal direction thanks to the slippery surface.

File Name: Supplementary Movie 6
Description: **Actuation of a droplet on vertically tilted surface.** The droplet can be actuated against gravity, thanks to the relatively large magnetic force.

File Name: Supplementary Movie 7
Description: **Programmed actuation of a droplet along a complex path.** Using programmed control of a series of electromagnets, the droplet can be guided to transport along a complex path, which is here a M-letter.

File Name: Supplementary Movie 8
Description: **Droplet splitting.** We designed a “ferrofluid-based cutting” method to split droplets, a basic operation of digital microfluidics. The detailed process can be found in Fig. 3a.

File Name: Supplementary Movie 9
Description: **Actuation of a droplet along a curved path with the help of field modulation layer.** To achieve complex-path actuation, we can also modulate the magnetic field distribution by incorporating a ferromagnetic field modulation layer. Here we used a layer with a curved ferromagnetic line, which drives a droplet placed directly above along this line. Another droplet placed away from the line was also driven, but less affected by the ferromagnetic line.

File Name: Supplementary Movie 10
Description: **Collective actuation of droplets using field modulation layer with an N-shape lattice pattern.** We can also use a patterned field modulation layer to facilitate the collective actuation of randomly distributed and random-sized droplets. In this way, these droplets were guided to coalesce and accumulated to defined positions. Here we used an N-shape pattern and the sprayed micro-sized droplets were accumulated to form an N-shape.
